# Finishing the Margins of Teeth

**Published:** 1891-11

**Authors:** 


ARTICLE V.

### FINISHING THE MARGINS OF TEETH.

In Illinois Society.

Dr. C. N. Johnson: We often find faulty preparation of proximal cavities in molars and bicuspids where they come to the grinding surfaces. They are left too narrow at the opening. The enamel should ordinarily be cut well back toward the cusps at the angel between the proximal and grinding surfaces. We thus avoid leaving a sharp edge of enamel which will eventually be broken down by mastication, and we provide better access to the cavity. The enamel can be trimmed away quickly and accurately with sandpaper disks, and I have found nothing that will answer so well in my hands. I can give the edge any shape I wish with them, and make the outline symmetrical and true. Of course, they will not do for the cervical margin—I refer to that part of the cavity approaching the grinding surface.

Dr. Black: I want to say a few words in regard to polishing margins of cavities with disks. Dr. Johnson may do this very nicely, but he may have unintentionally conveyed a wrong impression to some of the members by speaking of rounding the margins. I will ask you to stop and think what rounding margins means. If we polish the margins with anything that is soft, with any form of polishing powder used on soft material, on wood, rubber, or what not, we will round the margin. Suppose I have a cavity in a central incisor. Instead of cutting this square with the surface of the enamel, it should be a definite obtuse angle. If I polish it I round it over. It makes a a nice thing to fill against; but when I make a section of that tooth, including the filling made against such an edge, I will find that at the margin there is a feathered edge of gold. You will form a feathered edge of gold over it if you round the marginal edge of your enamel. You should have in your mind the form which will be given to the marginal edge of your gold filling, and thin it out to a feather edge in any case, for in the formation of a nice rounded margin to fill against you form an edge to your gold filling that is unreliable. In forming your marginal edges take a good, sharp chisel and plane them so as to make an obtuse, but definite angle of your filling, getting a proper beveled marginal edge of the enamel in every instance. It is just as important that the marginal edge of gold filling be right as the margin of the enamel itself. We must have them both right to get the best results. One of the difficulties in getting marginal edges of enamel in proper form is to prevent thinning out of the marginal edge of the filling material till it becomes unreliable. You should never polish the marginal edge of the enamel on which you are going to place a filling with pumice stone or paper disk unless it is in position in which you can so hold it to make a definite angle; then you need to be careful about it; never polish it with pumice carried by a stick or anything of this kind, but plane it with a good, sharp chisel, holding its edge parallel with the enamel rods, and the movement should be parallel with the marginal line of the cavity.

By the term marginal edge of the enamel I mean the cut edge of the enamel including its thickness. By the term enamel margin, or line of the enamel margin, I intend to convey the idea of the line forming the limits, or outline of the cavity. These two ideas should always be held distinct in the mind when discussing this subject. We form the line of the enamel margin by cutting away till the proper form is given to the outline of the filling. We form the marginal edge of the enamel by planting it to the proper bevel and smoothing it.

With the mesial surface of a molar tooth, with the cavity reaching nearly to the gingival line, we have the line of the enamel margin of the cavity, and make a bevel to form the marginal edge of that line of enamel margin. The line of the enamel margin is important, and the forming of the proper bevel on the marginal edge of the enamel is also important. This form is so important that we should be careful to make it with reference to the form of the marginal edge of the filling, as well as the marginal edge of the enamel.

We do not want any square edges of enamel, they should be beveled. We do not want an acute, or square corner.

When you form a cavity that is rounded and does not come close to the gingival line, the rule is you get recurrence of decay at this point, along the buccal or lingual marginal line of the filling near the gum. Even though you have made a perfect filling you get recurrence of decay. You get it a good many times when you do not make a perfect filling. The contact point is here and the gum covering the proximate surfaces of the teeth comes up and reaches that contact point in young persons in a normal condition. The interproximate space is filled with soft tissue, and every portion of tooth tissue that is covered by the gum septum is protected against decay; but in your operation you may have injured the gum septum, or it may have been injured in some other way so that it shrinks, and a considerable portion of the proximate surface of the tooth is exposed to the products of fermentation. Fermentation takes place between the two flat sides, hence we get a point of caries almost as soon as the shrinkage has taken place. Now, if you have prepared your cavity and rounded it here in the form of these cavities after simply removing the decay, and fill, the gum shrinks down. The part is exposed by the shrinkage of the gum, at the labial and lingual curves it approaches too near to contact to be self-cleaning, and we will get recurrence of decay. I find this recurrence on the incisors wherever there is a strong tendency to caries. Why? Because shrinkage of the gum has exposed these points to the action of the products of fermentation. Cut your cavity out toward the labial and lingual, so that it will be protected by the gum for the longest possible time. That is what I mean by extension for prevention. If you find corrosion of the surface of the enamel make the lines of the enamel margin include it, make extension further in the same direction to save the tooth from a recurrence of decay, extend the cavity around those angles as far as corrosion has occurred, then make a perfect filling, and it will be a long time before you have to fill that cavity again. It is better to do that in the first place than to remove the filling on account of recurrence of decay.

Dr. Cushing: I wish to say a word in reference to the method Dr. Black advocates, of handling the chisel, because I know a better margin can be made with a properly tempered cutting instrument, than by any other method, I do not care what it is. I am sure the margins can be made perfectly smooth, but the instruments must be keenly sharp, and that is something you rarely find dentists using; they attempt to use instruments that are not sharp, and that is the reason they do not make good edges by the use of cutting instruments. If the instruments are properly tempered, sharpened to the right bevel, kept keenly sharp, you can make a perfect edge.—*Review.*

---

